# Tightly-Coupled GNSS/Vision Using a Sky-Pointing Camera for Vehicle Navigation in Urban Areas

**DOI:** 10.3390/s18041244

**Published:** 2018-04-17

**Authors:** Paul Verlaine Gakne, Kyle O’Keefe

**Affiliations:** Position, Location and Navigation (PLAN) Group, Department of Geomatics Engineering, Schulich School of Engineering, University of Calgary, 2500 University Drive, N.W., Calgary, AB T2N 1N4, Canada; kpgokeef@ucalgary.ca

**Keywords:** visual odometry, upward-facing camera, motion estimation, satellites, GNSS, tightly-coupled integration, vehicle navigation, image segmentation, clustering algorithms

## Abstract

This paper presents a method of fusing the ego-motion of a robot or a land vehicle estimated from an upward-facing camera with Global Navigation Satellite System (GNSS) signals for navigation purposes in urban environments. A sky-pointing camera is mounted on the top of a car and synchronized with a GNSS receiver. The advantages of this configuration are two-fold: firstly, for the GNSS signals, the upward-facing camera will be used to classify the acquired images into sky and non-sky (also known as segmentation). A satellite falling into the non-sky areas (e.g., buildings, trees) will be rejected and not considered for the final position solution computation. Secondly, the sky-pointing camera (with a field of view of about 90 degrees) is helpful for urban area ego-motion estimation in the sense that it does not see most of the moving objects (e.g., pedestrians, cars) and thus is able to estimate the ego-motion with fewer outliers than is typical with a forward-facing camera. The GNSS and visual information systems are tightly-coupled in a Kalman filter for the final position solution. Experimental results demonstrate the ability of the system to provide satisfactory navigation solutions and better accuracy than the GNSS-only and the loosely-coupled GNSS/vision, 20 percent and 82 percent (in the worst case) respectively, in a deep urban canyon, even in conditions with fewer than four GNSS satellites.

## 1. Introduction

Autonomous vehicles rely on navigation sensors such as GNSS receivers, inertial navigation systems (INS), odometers, LiDAR, radar, etc. However, none of these sensors alone is able to provide satisfactory position solutions in terms of accuracy, availability, continuity and reliability all the time and in all environments. For example, INS are immune to interference. However, their performance degrades quickly when updates from other systems such as GNSS are not available. This is generally the typical scenario that is observed in deep downtown areas as presented in [1,2,3]. Moreover, integrating INS and GNSS might not be enough to ensure the availability of the position solutions with a certain accuracy and reliability. To avoid these kinds of situations, Gao et al. [4] proposed the integration of GNSS, INS and LiDAR. This is done to alternatively take advantage of the good performance of GNSS in open-sky and the rich LiDAR measurements in occluded outdoor environments for integration with the INS. The same reason drove [5] to combine GNSS, INS, an odometer and an omnidirectional camera. In open-sky scenarios (with no interference or spoofing), relying on GNSS for lane-level navigation is feasible. However, in challenging environments such as deep urban canyons, the GNSS signals are often blocked, reflected or shadowed. This significantly degrades the solution provided by the GNSS receiver. To overcome the disadvantages of GNSS in urban canyons, fusion with map data and other sensors is often employed (e.g., the Google self driving car [6]). However, combining sensors is expensive both in terms of cost and the computational load.

Besides sensors fusion, many multipath mitigation methods have been implemented in the literature, including: (i) antenna-based multipath detection/mitigation (antenna design [7], choke rings, controlled reception pattern antenna [8], angle of arrival [9], multiple antennas, dual-polarization antenna); (ii) receiver based (code discriminator design [10], early-late correlator comparison, Doppler domain, carrier smoothing, vector tracking, etc.); (iii) $C/N_{0}$-based multipath detection ($C/N_{0}$-based selection and weighting [11], multi-frequency $C/N_{0}$-based multipath detection, etc.); (iv) NLOS detection using a sky-pointing camera [12,13,14] and a 3D building model [15,16,17]. The research proposed in this paper uses a sky-pointing camera setup similar to what is used in [12,13,14]. The proposed method differs from the previous works in two ways. Previous works use a fish-eye camera in order to observe satellites at all elevations. However, low elevation satellites are not very beneficial for the position solution computation since most of the time they are corrupted by the tropospheric [18], ionospheric and multipath errors (causing the receiver to lose the lock on them frequently). Moreover, fish-eye cameras are not yet widely available on mass market products such as cellphones or car-mounted cameras. This paper uses a narrow field of view camera. This is practical since the method can be implemented on mass-market products that already incorporate such cameras. Furthermore, this setup helps to automatically exclude low elevation satellites. The second contribution regarding the multipath mitigation in this work is the segmentation algorithm proposed. A fast and robust segmentation algorithm tailored for navigation (with an upward-facing camera) is introduced.

Since almost all modern vehicles are equipped with multiple cameras, they constitute an attractive option for navigation. Some research has focused on using only the camera for navigation purposes. In [19], simultaneous localization and mapping (SLAM) methods are proposed using different camera configurations (monocular versus stereo), as well as different types of cameras (monocular/stereo visual spectral cameras versus RGB-D cameras). The proposed methods were tested on multiple publicly available datasets and showed consistency in terms of accuracy. An improved visual odometry and a robust visual localization using an omnidirectional camera are presented in [20,21], respectively. In the development of their improved omnidirectional visual odometry, the authors in [20,21] adapted the epipolar constraint to the omnidirectional camera sensor. They also improved the matching process by propagating the current uncertainty of the system. Overall, their method has proven to be robust because their adaptive matching process reduces false positives.

Another “stand-alone” solution available in the literature consists of using map data as presented in [22,23]. In order to facilitate the segmentation of the camera images into sky and non-sky, Petovello and He [23] use an infra-red camera, while a visual spectral camera is used in [22]. Moreover, Gakne and O’Keefe [22] proposed an improved image segmentation algorithm tailored for sky-pointing camera-based navigation. Practically, both methods consist of determining the vehicle position by matching skylines obtained from a 3D building model with the skylines observed with a camera mounted on the top of the vehicle. Using the skyline as a fingerprint reduces the vehicle localization problem to a place recognition problem. This approach could be useful in cases where it is difficult to obtain the position solution from any type of sensor since it can provide the user position even in the absence of GNSS. Readers can refer to the mentioned references for more details.

Integration between inertial measurement units (IMU) and cameras has also been extensively studied. A method of estimating robot attitude angles by fusing IMU data with a monocular vision system data is proposed in [24]. A six degrees of freedom (6-DOF) inertial sensor was fused with a low cost camera for an autonomous mobile robot navigation system using an extended Kalman filter. The proposed method has been shown to be effective in terms of computation load and attitude estimation accuracy.

More than two sensors can also be integrated. In [25], GNSS, INS and Ultra-Wideband (UWB) were integrated in a tightly coupled way using a robust Kalman filter that supported Vehicle to Infrastructure (V2I) communication. Furthermore, a monocular camera, IMU and GNSS were integrated for vehicle navigation in harsh environments in [26,27]. In [2], the navigation performance of GNSS/INS/vision integration was evaluated via simulated data, where the number of the observed satellites was continually decreased from three to one GPS satellites. As expected, the position error increased as the number of satellites decreased.

Finally, GNSS/vision integration has been presented in [28,29]. The vision system was combined with a differential GPS (DGPS) in [28]. The presented work focused on evaluating the heading obtained from the combined solution for micro-Unmanned Aerial Vehicle (UAV) navigation. Both simulated and field experiments showed that the DGPS/vision approach outperformed the fused onboard data (autopilot, magnetometer). A deeply-coupled GNSS/vision approach for vehicle navigation was introduced in [29]. The GNSS/vision system was able to provide continuous navigation in challenging environments that outperformed the GNSS/INS system.

The research proposed in this paper fits in this category with the contrast that this work uses only line-of-sight satellites selected by segmenting an upward-facing camera image into sky and non-sky areas. Moreover, our tightly-coupled implementation takes care of the scale factor issue that affects monocular vision systems.

It should be noted that the above-mentioned approaches do not represent an exhaustive list of sensor fusion approaches that exist in the literature. An autonomous car such as Faraday’s FF 91 fuses more than 30 sensors including cameras, radars and ultrasonic sensors. This list of approaches is given for illustration of what exists in the literature pertaining to the integration of optical sensors with various other types of sensors.

The objective of this paper is to evaluate the performance of a system that integrates monocular visual odometry (implemented with a camera pointing upward) and GNSS signals. Since GNSS signals are subject to severe multipath in urban canyons, an image-based multipath mitigation approach was developed. This approach consists of segmenting an image into sky and non-sky areas. Images taken with an upward-facing camera are complex and difficult to segment, we introduce a tailored segmentation method appropriate for vehicle navigation applications.

The remainder of this paper is organized as follows: Section 2 briefly presents the existing works related to this research; Section 3 describes the vehicle motion estimation steps using a camera; Section 4 implements the improved segmentation approach, as well as the Non-Line-Of-Sight (NLOS) mitigation algorithm; Section 5 presents the GNSS/vision integration strategy we propose; Section 6 describes the experiment, results and analysis; and Section 7 draws some conclusions.

## 2. Background and Related Works

The research presented in this paper involves many fields including image processing and computer vision, GNSS, as well as estimation for navigation (Kalman filtering).

The image-based NLOS satellites exclusion used in this research heavily relies on the accuracy of the segmentation algorithm [14,30,31]. Let us assume that due to a segmentation error, a building is classified as sky. A satellite that is projected in that area will not be excluded, i.e., will be considered as a Line-Of-Sight (LOS) satellite (but actually it is not). Given that even a single GNSS signal affected by multipath can cause a significant position error, this could significantly degrade our GNSS-only solution. Few segmentation algorithms have been evaluated in the literature [12,14]. In Attia et al. [12], a set of supervised and unsupervised (pixel) classifiers were compared, and it was concluded that the proposed method (i.e., the Geodesic Reconstruction by Dilatation (GRD) coupled to the Fisher clustering algorithm) was more efficient than the other evaluated classifiers. However, the Fisher classifier takes about 1.03 s to process one image. This is computationally heavy compared to the method proposed in [14], where it was found that the Otsu method outperforms the other considered algorithms (Meanshift, HMRF-EM, graph-cut) for this specific upward-pointing camera application. The Otsu method segments an image with a resolution of 1288×728 in 0.015 s and provides an accuracy of 94.7%. To increase the accuracy of the segmentation algorithms, Petovello et al. and Meguro et al. [23,30] used infrared cameras. However, to date, these types of cameras are not yet available on mass market devices. This leads us to choose a visual spectrum standard camera with a narrow Field Of View (FOV) and suggests a need to improve the segmentation algorithm. A fast and accurate segmentation method is presented in Section 4.

Obtaining the position of a robot or vehicle using visual features is one of the most studied problems in the computer vision community. The subject is extensively discussed in [32]. There are several approaches that are usually classified based on how feature points are matched. These include 2D-to-2D matching, 3D-to-3D and 3D-to-2D [32]. An example of monocular visual odometry using 3D-to-2D matching is presented in [33] where a database is used to store a set of point clouds (feature database) registered with intensity information. 2D features are detected in an image captured by the camera, and the match is done with the content of the database. Then, by applying the PnP algorithm, the camera’s rotation and translation are obtained. In contrast, in the present paper, we propose a 2D-to-2D approach where 2D feature points are detected on successive images and matching and tracking is used to obtain the position of the camera with respect to the tracked features. Other references such as [20,34,35,36] integrate visual odometry into SLAM applications. This not only provides an advantage of allowing the methods to be used for multiple purposes (positioning, navigation and mapping) but also increases the robustness of these methods. In contrast, in this work, visual odometry based on feature point detection, matching and tracking is used since there is no need in our application (so far) to build a map as the vehicle navigates.

GNSS fundamentals are highly documented in the literature [37]. In open-sky, GNSS performs well. However, in harsh environments, it performs poorly. In such areas where some weak GNSS signals are available, high sensitivity receivers using massive numbers of parallel correlators and longer integration times can be used. However, this approach involves higher processing, complexity and power costs [38,39]. Different GNSS receiver architectures that can improve the receiver performance are also introduced in [40,41].

Many works related to GNSS/vision vary from the type of the measurements used (pseudorange versus carrier phase) or the type of the integration (EKF, loosely coupled, tightly coupled or ultra-tightly coupled). Some examples of these types of integration can be found in [2,29,42,43]. In this work, selected line-of-sight satellite measurements are tightly integrated with the visual odometry. The satellite selection, discussed previously, helps to reduce the multipath effect, and tightly coupling the selected satellites with the vision ensures that the GNSS measurements are used even if fewer than four line-of-sight satellites are available.

## 3. Vehicle Motion Estimation Using an Upward-Facing Camera

This section discusses the steps required for vehicle ego-motion estimation.

### 3.1. Camera Calibration and Image Rectification

Similar to most computer vision applications, camera calibration and image rectification are two important steps in the development of the proposed method. The calibration method described in [44] is used in this paper as implemented in the OpenCV library [45]. It consists of determining the camera matrix, as well as the distortion coefficient matrix. In fact, there are two main lens distortions: the radial and the tangential distortions. The former are caused by the shape of the lens, while the later arise from manufacturing defects resulting from the lens not being exactly parallel to the imaging plane. Concerning the radial distortion rectification, given a current distorted pixel $\tilde{p}$ with coordinates $\left(\tilde{x},\tilde{y}\right)$, the radial location of $\tilde{p}$ will be rescaled [46] on the undistorted output image as:

(1) $$x=\tilde{x}\left(1+r_{1}d^{2}+r_{2}d^{4}+r_{3}d^{6}\right)$$

(2) $$y=\tilde{y}\left(1+r_{1}d^{2}+r_{2}d^{4}+r_{3}d^{6}\right)$$

where:

$r_{i\;(i=1,2,3)}$ denotes the radial lens distortion parameters;
*x* and *y* are the new coordinates of the pixel as a result of the correction;
$d=\sqrt{{\left(\tilde{x}-x_{c}\right)}^{2}+{\left(\tilde{y}-y_{c}\right)}^{2}}$ is the distance of the distorted coordinates to/from the principal point. $x_{c}$ and $y_{c}$ are the coordinates of the principal point.

Regarding the tangential distortion, it is corrected by using:

(3) $$x=\tilde{x}+2t_{1}\tilde{x}\tilde{y}+t_{2}\left(d^{2}+2{\tilde{x}}^{2}\right)$$

(4) $$y=\tilde{y}+t_{1}\left(r^{2}+2{\tilde{y}}^{2}\right)+2t_{2}\tilde{x}\tilde{y}$$

where $t_{j\;(j=1,2)}$ are the tangential distortion parameters.

Besides the lens distortion correction, the camera focal length f and the principal point coordinates $\left(x_{c},y_{c}\right)$ are determined to build the camera matrix. More details can be found in [46]. The algorithm successively estimates the parameters using the closed-form solution [44] and maximum-likelihood estimation using all detected corners on a chessboard in multiple views. It minimizes the error between the projected object space coordinates on the image with the estimated parameters and the measurements of the feature points [29], using the Levenberg–Marquardt algorithm [47]. Once the image is rectified and the intrinsic parameters of the camera obtained, further processing can be carried out.

### 3.2. Feature Detection, Description and Matching

Feature extraction and matching constitute essential processes for the platform (robot, vehicle) motion estimation. In this work, we use ORB (Oriented FAST and Rotated BRIEF) [48] as the feature detector and descriptor algorithm. ORB combines an improved version of the Feature from the Accelerated Segment Test (FAST) algorithm [49] for feature detection, with the Binary Robust Independent Elementary Features (BRIEF) [50] descriptor extraction process. Its main advantages are its low run-time and its ability to detect a relatively large number of features.

#### 3.2.1. Feature from Accelerated Segment Test Feature Detector

**Figure 1:**
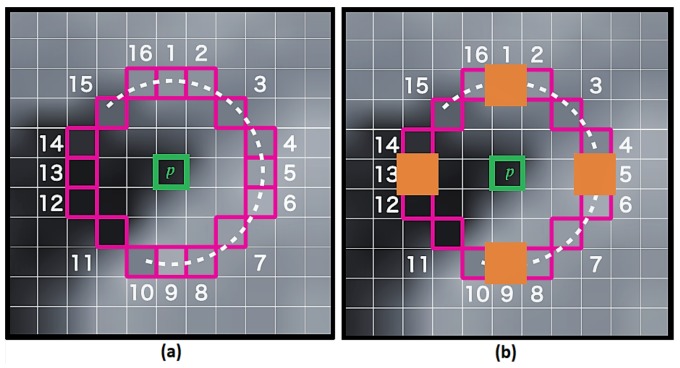
FAST feature detection illustration. (**a**) *p* is the candidate corner; pink-highlighted squares are sixteen pixels used in the feature detection; the dashed line passes through twelve consecutive pixels that are brighter than the pixel at *p*. (**b**) Same as (a) with the four pixels at Indexes 1, 5, 9 and 13 filled in dark orange used to speed up the threshold test.

FAST considers a pixel at the centre of a candidate corner *p* and performs a test on a circle of sixteen pixels around *p* as shown in Figure 1a. If twelve of those pixels are brighter than the pixel at the centre of the candidate point, then *p* is a corner. To speed up the processing, FAST starts by setting a threshold intensity value T (e.g., 20% of the intensity of *p*). Then, it compares the intensity of the pixels at Indexes 1, 5, 9 and 13, as highlighted in Figure 1b with T. If at least three out of four of these pixels’ intensities are above or below the threshold (threshold test), the test continues with the other sixteen pixels, and at least twelve of these pixels intensities should satisfy the threshold test to declare *p* as an interest point. If at the first level there are not at least three (out of four) pixels’ intensity satisfying the threshold test, FAST rejects the current candidate corner. Despite its computation efficiency, FAST features do not have an orientation component. The enhanced FAST used in the ORB algorithm addresses this issue by using the intensity centroid [48]. This method uses the vector representing the offset of a corner intensity from its centre. The enhanced FAST uses this vector to impute the orientation information.

#### 3.2.2. Binary Robust Independent Elementary Features Descriptor

The BRIEF algorithm described in [50] is not designed to be rotationally invariant and tolerates small amounts of rotation only. In other words, if two images of the same viewpoint are taken with a large camera rotation (≳$10^{\circ }$), the feature description using BRIEF will fail. Indeed, given *n* (n=128,256 or 512) predefined pixel pairs within an image patch, BRIEF uses local binary tests to produce *n*-bit strings. A so obtained vector representation is very sensitive to rotation variation. Thus, ORB uses a rotation-invariant version of BRIEF (rBRIEF (rotation-aware BRIEF)) presented in [48].

After applying the FAST and BRIEF algorithms, the Hamming distance [51] is used to match the obtained strings of descriptors. However, false matches might happen between certain very similar features in the viewpoints. These types of mismatches can cause severe ego-motion estimation error. Thus, the outliers need to be removed.

### 3.3. Outlier Rejection

Due to the dense traffic in the urban canyons, clutters and moving objects such as cars or pedestrians are captured by the camera. The detected corners on these objects will be considered by the tracking algorithm as fixed points, while in reality, the features are dynamic. This constitutes the major source of outliers in urban areas where humans move actively. Although our configuration (with a sky-pointing camera) is beneficial in the sense that it will capture very few moving objects (depending on the FOV of the camera), in order to make the developed method camera configuration independent, the random sample consensus (RANSAC) algorithm [52] is used. The advantage of this method is its robustness when facing a high number of outliers, as well as its low computational load. RANSAC randomly selects a subset of feature points from the full set of the tracked features. The non-considered feature points are then reprojected using the estimated states. The errors between the measured image coordinates and the computed reprojection coordinates are tested using the specified measurement accuracies, the estimated state accuracy, as well as the defined confidence level. The process is repeated to refine the remaining feature points that are roughly consistent with the previous estimates. The inliers are those feature points that are consistent, and the outliers are those that are not. This procedure is repeated a fixed number of times.

### 3.4. Motion Estimation Process

Once the correspondences are established between the detected features, the next step is the estimation of the essential matrix E. The matrix E represents the geometric relations between two consecutive images $I_{k-1}$ and $I_{k}$. It contains the camera motion parameters up to an unknown scale factor for the translation [32] in the form:

(5) $$E_{k}≃{\hat{t}}_{c_{k}}r_{c_{k}}$$

where:

*k* denotes the image frame number;
${\hat{t}}_{c_{k}}=\left[\begin{array}{ccc} 0 & -t_{z} & t_{y}\\ t_{z} & 0 & -t_{x}\\ -t_{y} & t_{x} & 0 \end{array}\right]$;
$r_{c_{k}}$ is the rotation matrix;
and $t_{c_{k}}={\left[\begin{array}{ccc} t_{x} & t_{y} & t_{z} \end{array}\right]}^{T}$, where $t_{x}$, $t_{y}$ and $t_{z}$ are the relative translations following the camera axes.

Equation (5) is resolved based on the five-point algorithm solver [53], which provides a certain robustness in the presence of outliers.

The camera translation up to a scale, as well as its rotation are thus obtained for two consecutive images and can be used in the subsequent steps in our integration algorithm. One of the most important properties of the motion estimation in this paper is the epipolar constraint that can be described by the essential or the fundamental matrix between two camera positions. The epipolar constraint provides that the projection of a point on the actual image frame $I_{k}$ must fall in the intersection of an epipolar plane with the image plane [53,54]. By this process, it is possible to verify if two 2D feature points correspond to the same 3D point. Thus, the epipolar constraint determines the line on which the feature point on the image $I_{k}$ falls in the image $I_{k-1}$. This constraint is formulated as:

(6) $${p^{\prime }}^{T}Ep=0$$

**Figure 2:**
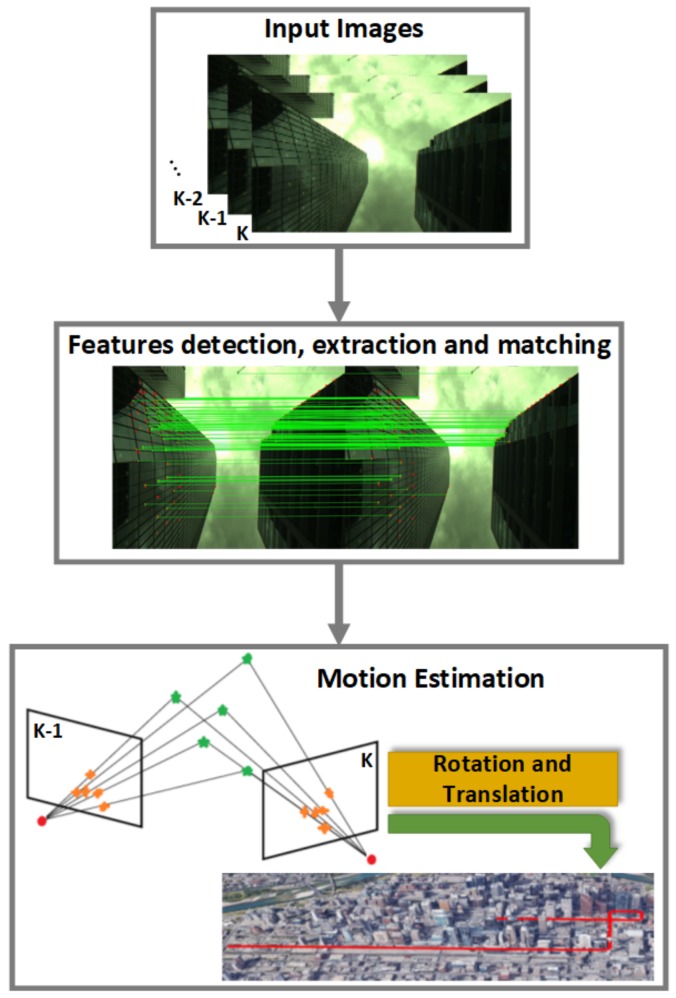
Feature points-based visual odometry.

The step by step determination of the essential matrix is presented in [53]. Once E is estimated from the matched feature points, the relative rotation and translation of the camera are recovered by using the cheirality check [53]. The essential matrix is first decomposed using Singular Value Decomposition (SVD) [54] to obtain four possible poses (rotation/translation), then the verification of the possible pose hypotheses by doing the cheirality check is performed. The cheirality check consists of verifying that the feature point lies in front of the camera (i.e., the triangulated feature point should have positive depth). By this process, the correct rotation $r_{c}$ and translation $t_{c}$ can be identified and used for the GNSS and vision integration (see Section 5). However, as mentioned previously, using a monocular system, the magnitude of the translation between two consecutive images suffers from scale ambiguity. The integration with GNSS helps to overcome this issue. The visual odometry steps [55] are depicted in Figure 2.

### 3.5. Computing the Rotation and Translation Using the Singular Value Decomposition Approach

Given two sets of feature points $f_{p_{1}}=\left\{p_{0},p_{2},\ldots ,p_{M-1}\right\}$ and $f_{p_{2}}=\left\{p_{0}^{\prime },p_{2}^{\prime },\ldots ,p_{M-1}^{\prime }\right\}$ (*M* is the total number of feature points), the process described here consists of determining the rotation and translation based on the least squares approach using the SVD. Having the sets of the feature points, we are looking for the rotation $r_{c}$ and translation $t_{c}$ such that:

(7) $$\left(r_{c},t_{c}\right)=\arg\min_{r_{c_{min}},t_{c_{min}}}\sum_{j=0}^{M-1}w_{j}\left|\right|\left(r_{c_{min}}p_{j}+t_{c_{min}}\right)-p_{j}^{\prime }{\left|\right|}^{2}$$

where $w_{j}>0$ is the weight of each point pair.

The algorithm for computing $r_{c}$ and $t_{c}$ is summarized in Algorithm 1. More details can be found in [56].

From the rotation matrix, the pitch, roll and yaw of the platform can be determined by a simple conversion of the rotation matrix to Euler angles (for example). It should be noted that, instead of using the visual odometry to determine the platform orientation, the concept of vanishing points can also be used. This is done in [55,57].
**Algorithm 1:** Rotation and Translation Computation Algorithm.**Input** : $f_{p_{1}}=\left\{p_{0},p_{2},\ldots ,p_{M-1}\right\}$ and $f_{p_{2}}=\left\{p_{0}^{\prime },p_{2}^{\prime },\ldots ,p_{M-1}^{\prime }\right\}$
 **Output**: $r_{c}$ and $t_{c}$
 *// initialization*
 $r_{c}=I_{3\times 3}$
 $t_{c}=0_{3\times 1}$
 *// iterate to the total number of feature points*
 
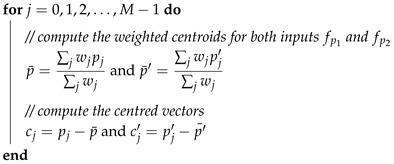

 *// compute the covariance matrix*
 $\mathrm{Cov}=C\;\times \;diag\left(w_{0},w_{1},\ldots ,w_{M-1}\right)\;\times \;{C^{\prime }}^{T}$, C and $C^{\prime }$ = matrices with $c_{j}$ and $c_{j}^{\prime }$ as their columns  *// determine the SVD of Cov*
 *$\mathrm{Cov}=U\Sigma V^{T}$*
 *// compute the rotation*
 $r_{c}=V\left[\begin{array}{ccccc} 1 & & & & \\ & 1 & & & \\ & & \ddots & & \\ & & & 1 & \\ & & & & \det\left(VU^{T}\right) \end{array}\right]U^{T}$
 *// compute the translation*
 $t_{c}=\bar{p^{\prime }}-r_{c}\bar{p}$
 **return:**
$r_{c}$ , $t_{c}$

## 4. Camera-Based Non-Line-Of-Sight Effect Mitigation

This section presents the vision-based method we used to reduce and mitigate the NLOS GNSS effects.

### 4.1. Image Segmentation-Based NLOS Mitigation Algorithm

**Figure 3:**
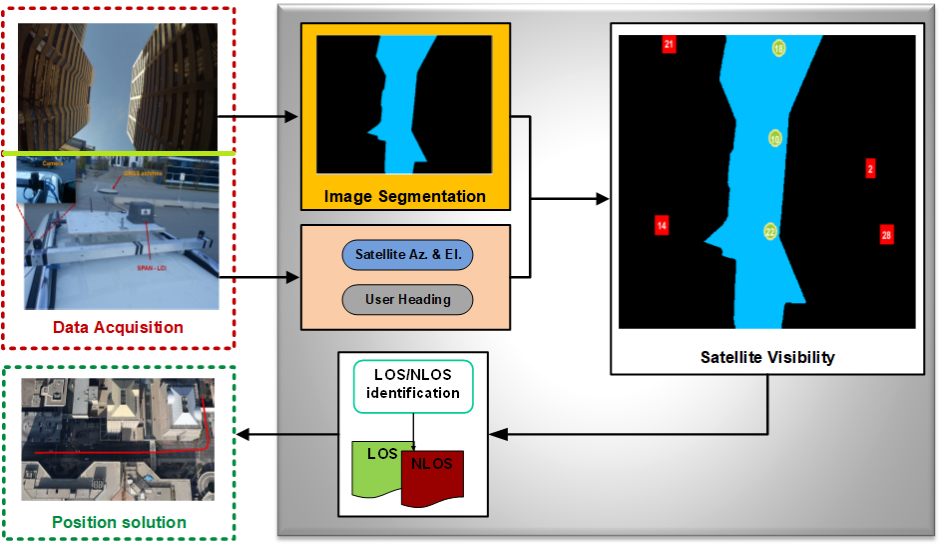
NLOS effect mitigation algorithm steps: “Data Acquisition” box: synchronized GNSS receiver and monocular system (down) and an example of the captured image (top); “Image Segmentation” box: the blue area in the segmented image represents the sky and the black area is the non-sky area (obstacle); “Satellite Visibility” box: the green circles are the non-obstructed satellites, and the red rectangles are the obstructed satellites that will be ignored.

Image segmentation is a process that consists of partitioning the image into two or more portions called segments. The resulting output image is a simplified version of the input image that depicts “meaningful” regions or objects only. The NLOS mitigation method used in this paper consists of detecting and rejecting the obstructed satellites that are the principal sources of the multipath and NLOS errors. Our method uses a sky-pointing camera to acquire the GNSS receiver antenna’s surrounding environment [14,31,58]. The captured images need to be segmented into sky and non-sky. The tracked satellites are then reprojected onto the segmented image. Finally, the satellites that lie on the non-sky areas in the image are removed before computing the final position solution. The concept has been presented in our previous work [14] where we evaluated various segmentation algorithms in different weather conditions. The steps of this method are depicted in Figure 3.

**Figure 4:**
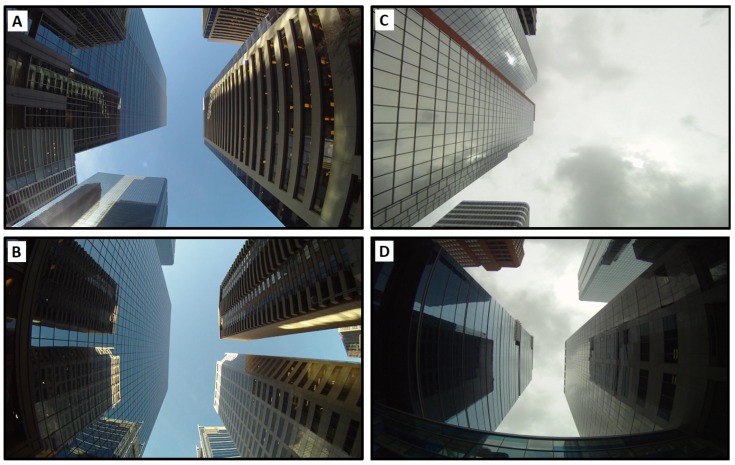
Example of complex downtown images taken using an upward-facing camera. Correct image segmentation can be affected by the environment illumination, building facades (reflections observed on the glasses facades). Left (**A**,**B**): sunny weather condition; right (**C**,**D**): cloudy weather condition.

However, segmenting an image into sky and non-sky is not an easy task due to lighting variations, weather conditions and the facades of buildings (e.g., buildings with glass). Figure 4A,B,D, shows few challenging images to segment (in sunny and cloudy weather conditions).

**Figure 5:**
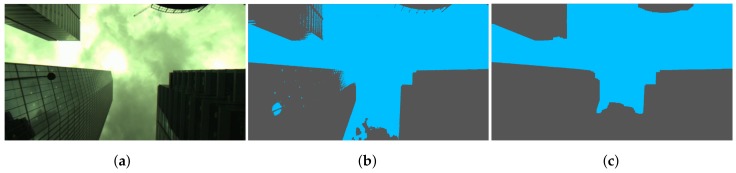
Image segmentation using the Otsu methods. (**a**) Original; (**b**) Otsu; (**c**) Improved Otsu [14].

Using conventional image segmentation algorithms, most of them initially developed for different purposes than outdoor navigation (e.g., biomedical applications), will produce poor results. Figure 5 shows the Otsu method segmentation applied to binarize an image. Figure 5b shows the Otsu method without pre-processing. Figure 5c shows the Otsu method with pre-processing that we introduced in [14].

It can clearly be seen that accurately segmenting this image is challenging, and segmentation errors are observed both in Figure 5b and Figure 5c. Surrounding buildings and sky are reflected and visible on the buildings with glass facades. Partitioning such images in a way that pixels belonging to sky and buildings share the same label with regard to features such as colour or texture is generally difficult to achieve.

**Figure 6:**
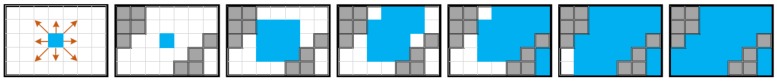
Flood-fill algorithm illustration. Pixels are filled from the centre of the image until an edge is reached following the eight arrows indicated in the first (left-most) box.

In this work, we introduce an improved segmentation approach tailored for vision-based outdoor vehicular navigation applications. The algorithm consists of using edges in order to partition an image into sky and non-sky segments. Objects (buildings, sky, trees, etc.) in an image visually occupy regions. These regions are separated by using algorithms such as the canny edge detector [59]. The centre of the image is assumed to be a sky segment. This assumption makes sense, since in normal conditions, a sky-pointing camera mounted on the top of a land vehicle driven on the road will not be inclined in a such way that the centre of the image is obstructed by a building. From the middle of the image, the following pixels are “filled” as sky until we reach an edge. Then, anything beyond the edge is non-sky. However, as shown in Figure 4C, clouds (or sun in some other cases (not shown)) can cause the sky area to appear non-homogeneous. The canny edge detector in this case will detect edges within sky area and thus leads to segmentation errors. To avoid these kinds of issues, the developed algorithm includes four steps:

- Image filtering: Given that the images captured using a sky-pointing camera are highly corrupted by bright (e.g., sun light) and dark (e.g., buildings or clouds) objects/structures, we adopted a sequential application of open-close filters denoted Alternate Sequential Filter (ASF) [60]. Indeed, we observed that when the noise is wide-spread over an image, using a single open-close filter with a large structuring element leads to segmentation errors (bright objects tend to be lost and the difference between the sky segment and the other structures in the image becomes hard to observe). ASF provides efficient results since it alternates openings and closings proceeding in an incremental way from small to a given size of the structuring element *m*, m≥1 [60]. Consider $\Omega_{m}$ and $\varsigma_{m}$ the morphological opening and closing of size *m*, respectively. The ASF is a sequential combination of $\Omega_{m}$ and $\varsigma_{m}$ such as $\gamma_{m}=\Omega_{m}\varsigma_{m}$ is a morphological filter. Thus, we have:
  (8) $$ASF_{m}=\gamma_{m}\ldots \gamma_{2}\gamma_{1}$$
  For illustration, if m=2, we have $ASF_{2}\left(I\right)=\Omega_{2}\left[\varsigma_{2}\left(\Omega_{1}\left[\varsigma_{1}\left(I\right)\right]\right)\right]$, where *I* is the image to filter. The result obtained by using the filter defined in Equation (8) is a less noisy image than the original image. The different portions of the image are more obvious after this step. However, the output of the ASF still provide edges within the sky areas. Since our approach uses edges to categorize sky and non-sky, it is important to remove such edges. For this reason, the levelling algorithm [61] is used along with ASF to find a good trade-off between good identification of edges between sky and buildings and suppression of edges within the same structures (sky and other objects in the image).
- Colour space conversion: once the image is filtered, we determine that the perceptual brightness (luminance) of the image is enough to accurately distinguish the main partitions contained in the image since it depicts sharp details. For this reason, the RGB (Red, Green and Blue) image is converted to the Luv colour space. The luminance channel L is then extracted for further processing.
- Edge detection: the luminance channel extracted from the filtered image is smooth and suitable for edge detection with limited errors. The edge detection here consists of finding discontinuity in the luminance of the pixels within the image. The well-known canny edge detector [59], which consists of smoothing the image with a Gaussian filter, computing horizontal and vertical gradients, computing the magnitude of the gradient, performing non-maximal suppression and performing hysteresis thresholding, is used in this paper.
- Flood-fill algorithm application: At this point, the decision should be made on which edges mark the limit between sky and non-sky areas. The flood-fill step is initialized by assuming the pixel at the centre of the image as belonging to the sky category. Then, the pixels from the centre of the image are filled until we reach an edge. In other words, we used the output of the edge detector algorithm as a mask to stop filling at edges. This process is illustrated in Figure 6.

**Figure 7:**
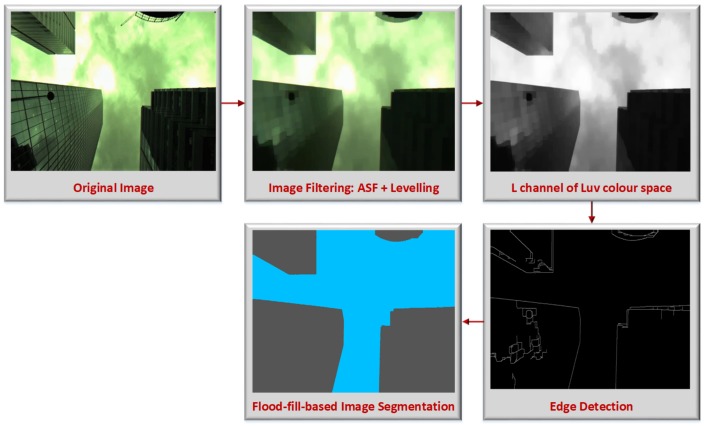
Flood-fill-based image segmentation steps. Following the arrow direction we have the following: first box: original image; second box: filtered RGB image; third box: luminance channel extracted from the RGB to Luv colour space conversion; fourth box: detected edges; fifth box: image segmentation result.

Figure 7 shows the steps of the image segmentation algorithm we implemented. The original image is a relatively complex image containing sun light, clouds, reflective building facades, etc. The second box represents a smoother (filtered) image. The small bright spots observed on the building facades are filtered out, and structures look sharper and less influenced by the sun. The third box depicts the luminance channel of the filtered image represented in the Luv colour space. Dominant segments are kept, and lighting effects decrease, while less dominant segments look smoother. This reduces/eliminates edges within the same structures, which leads to accurate edge detection between segments, as depicted in the fourth box. Accurate edge detection naturally leads to accurate image segmentation (fifth box). In the segmented image (fifth box), the sky is represented in blue colour, and the non-sky or obstacles are represented in dark grey colour.

### 4.2. Satellite Projection and Rejection

The method used in this paper consists of projecting all the tracked satellites onto the successive images and classifying them into LOS satellites (in the sky area) and NLOS satellites (in non-sky area). To project the satellites on an image, we need:

- The distance from the centre of the image in pixels ($d_{c_{\mathrm{pix}}}$): this corresponds to the elevation angle of the satellite ($\varepsilon_{sat}$),
- The azimuth within an image: for this, the heading of the platform is required.

**Figure 8:**
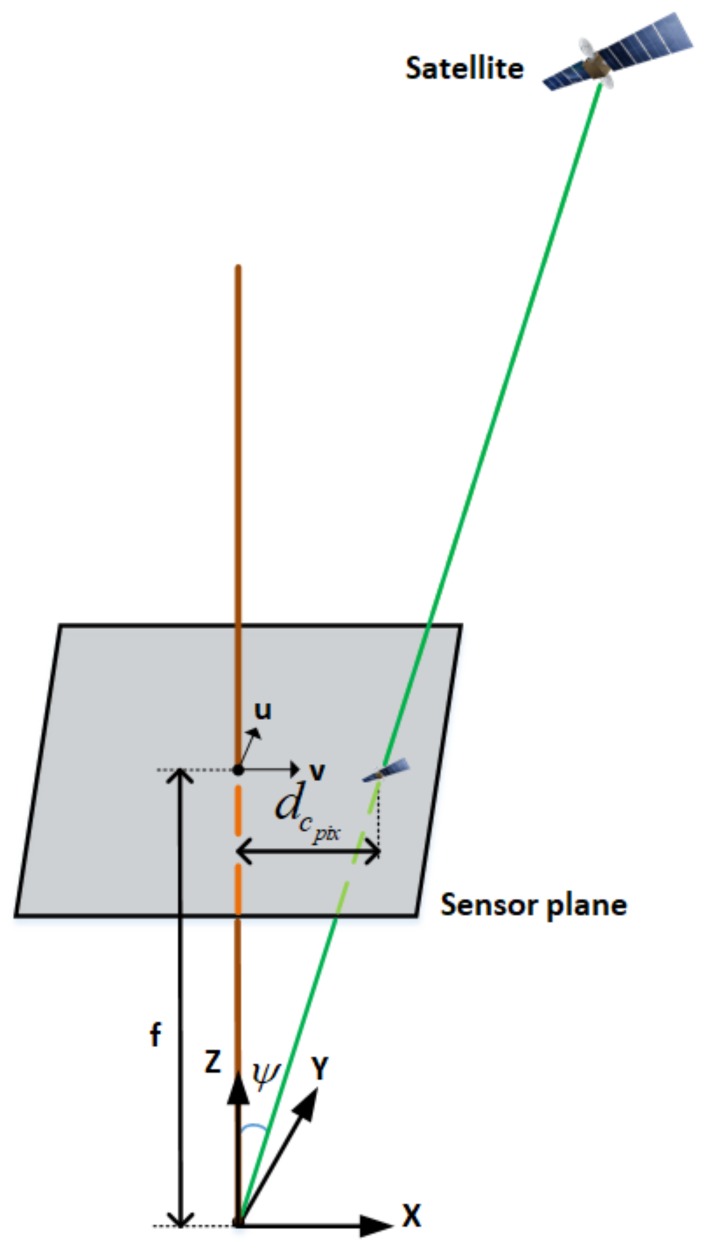
Modelling of the satellite projection on the image.

The geometry shown in Figure 8 is used to determine the distance ($d_{c_{\mathrm{pix}}}$, in pixels) from the centre of a calibrated image to a satellite’s projection onto the image plane.

To determine $d_{c_{\mathrm{pix}}}$ corresponding to $\varepsilon_{sat}$, we defined a mapping function, which is a function of the focal length **f** and the angle Ψ (see Figure 8). Assuming that the optical centre is constantly zenith pointing, the angle Ψ is defined as:

(9) $$\Psi =\frac{\pi }{2}-\varepsilon_{sat}$$

Having a calibrated camera and from Figure 8 and Equation (9), we obtain:

(10) $$d_{c_{\mathrm{pix}}}=f_{\mathrm{pix}}\cdot \tan\left(\frac{\pi }{2}-\varepsilon_{sat}\right)$$

where $f_{\mathrm{pix}}$ is the focal length in pixels.

Thus, given the centre of the image coordinates ($x_{c}$,$y_{c}$), the platform heading, as well as the satellite azimuth, the coordinates of the projection of the satellite on the image (in pixel) are given by Equations (11a) and (11b) below:

(11a) $$\begin{array}{c} x_{Sat_{\mathrm{Img}}}=x_{c}+d_{c_{\mathrm{pix}}}\cdot \cos\left(\alpha_{\mathrm{platform}}+\alpha_{sat}\right) \end{array}$$

(11b) $$\begin{array}{c} y_{Sat_{\mathrm{Img}}}=y_{c}-d_{c_{\mathrm{pix}}}\cdot \sin\left(\alpha_{\mathrm{platform}}+\alpha_{sat}\right) \end{array}$$

where:

$\left(x_{Sat_{\mathrm{Img}}},y_{Sat_{\mathrm{Img}}}\right)$ are the projected satellite coordinates on the image plane;
$\alpha_{\mathrm{platform}}$ is the heading of the platform;
$\alpha_{sat}$ is the satellite azimuth.

**Figure 9:**
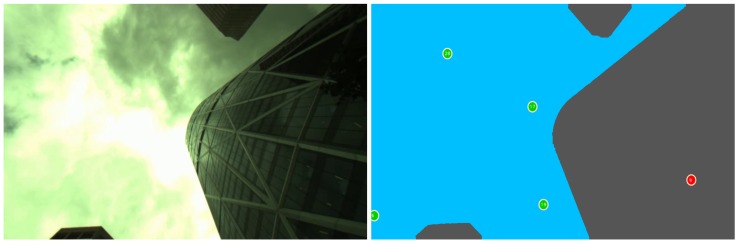
Example of satellites’ classification based on their obstruction status. Left: original image; right: satellites projection and classification. Obstructed, NLOS (PRN9), and non-obstructed, LOS (PRN6, PRN17, PRN19, PRN28), satellites are represented in red and green, respectively.

An example of the satellite projection result at a given vehicle location is depicted in Figure 9. The non-obstructed satellites, also called LOS satellites, are those that lie in the sky area represented with the green circles. In Figure 9, they are PRN6, PRN17, PRN19 and PRN28. The obstructed satellite (NLOS satellite, red circle) in this case is PRN9.

Thus, at this specific epoch, PRN9 will be rejected, and PRNs 6, 17, 19 and 28 will be used for further processing.

## 5. Algorithm Development: GNSS/Vision Integration

**Figure 10:**
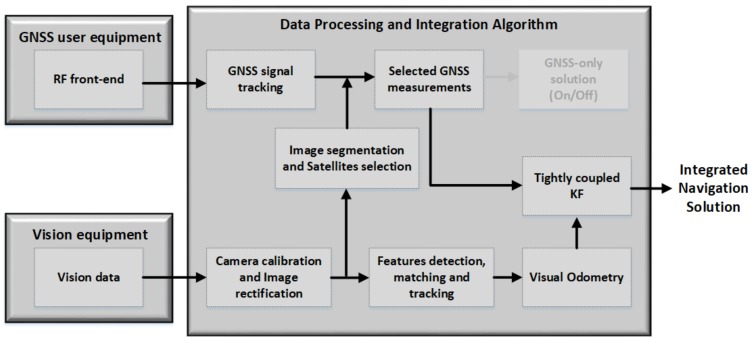
The proposed GNS/vision tightly-coupled integration’s general architecture.

In this paper, we are proposing the integration of the GNSS raw measurements with the vision motion estimation in a tightly-coupled manner. The proposed approach diagram architecture is depicted in Figure 10.

The acquired images (from the vision equipment box) are rectified and used in two different ways: firstly, the images are not modified in order to implement the visual odometry and obtain the camera pose, as well as its relative position; secondly, the “copy” of these images is processed (segmented following the steps presented in Figure 7) to implement the multipath mitigation algorithm. Only the selected satellites’ (LOS satellites) raw measurements are used in the filter.

### 5.1. Global Navigation Satellite Systems

From all the GNSS measurements [37] available, in this paper, we use the pseudorange and pseudorange rate (Doppler). The carrier phase and the accumulated delta range are left for future work.

### 5.2. Pseudorange Observation

The pseudorange observation between the user and a satellite is related to the user position and clock states as:

(12) $$\rho_{i}=|r_{i}-r_{u}|+\;cdt+\mu_{\rho_{i}}$$

where:

$\rho_{i}$ is the pseudorange of the $i^{\mathrm{th}}$ satellite;
$r_{i}$ denotes the $i^{\mathrm{th}}$ satellite’s position at the transmission time;
$r_{u}$ represents the user position at the reception time;
cdt is the receiver clock bias;
$\mu_{\rho_{i}}$ denotes the sum of all errors on the measurement;
∣•∣ denotes the magnitude of a vector.

Given an a priori estimate of the state, an estimate of the clock bias and the errors, the estimate of the pseudorange measurement is given by:

(13) $${\hat{\rho }}_{i}=|r_{i}-{\hat{r}}_{u}|+\;c\hat{dt}+{\hat{\mu }}_{\rho_{i}}$$

where all $\hat{\left(\cdot \right)}$ represent the corresponding estimates of the parameters as defined in Equation (12).

Thus, the residual is expressed as:

(14) $$\delta \rho_{i}=\rho_{i}-{\hat{\rho }}_{i}=\left[\begin{array}{cc} {\hat{1}}_{i}^{T} & 1 \end{array}\right]\left[\begin{array}{c} r_{u}-{\hat{r}}_{u}\\ c(dt-\hat{dt}) \end{array}\right]+\mu_{\rho_{i}}-{\hat{\mu }}_{\rho_{i}}$$

where ${\hat{1}}_{i}^{T}=\frac{r_{i}-{\hat{r}}_{u}}{|r_{i}-{\hat{r}}_{u}|}$ is the unit vector from the estimated user position to the satellite.

Likewise, the pseudorange rate observation is given by the following:

(15) $${\dot{\rho }}_{i}=\left(\nu_{i}-\nu_{u}\right)\;•\;\frac{r_{i}-r_{u}}{|r_{i}-r_{u}|}+d+\mu_{{\dot{\rho }}_{i}}$$

where:

$\nu_{i}$ and $\nu_{u}$ are the ith satellite and user velocities, respectively, expressed in the Earth-Centred Earth-Fixed (ECEF) coordinate frame;
*d* is the receiver clock drift in m/s;
$\mu_{{\dot{\rho }}_{i}}$ represents the ensemble of errors in the measurement in m/s;
(•) denotes the dot product.

The estimate of the pseudorange rate is expressed as:

(16) $$\hat{\dot{\rho_{i}}}=\left(\nu_{i}-{\hat{\nu }}_{u}\right)\;•\;{\hat{1}}_{i}+\hat{d}+{\hat{\mu }}_{{\dot{\rho }}_{i}}$$

where $\hat{\left(\cdot \right)}$, as previously, represents the estimates of the corresponding parameters.

From Equations (15) and (16), we obtain the linearized pseudorange rate measurement given by the equation:

(17) $$\delta \dot{\rho_{i}}={\dot{\rho }}_{i}-\hat{\dot{\rho_{i}}}=\left[\begin{array}{cc} -{\hat{1}}_{i}^{T} & 1 \end{array}\right]\left[\begin{array}{c} \nu_{u}-{\hat{\nu }}_{u}\\ d-\hat{d} \end{array}\right]+\mu_{{\dot{\rho }}_{i}}-{\hat{\mu }}_{{\dot{\rho }}_{i}}$$

From the above equations, the set of GNSS observations ($z_{Gnss}$) is used in the form of measurements:

(18) $$\delta z_{Gnss}=\left[\begin{array}{c} \delta \rho_{0}\\ \delta \rho_{1}\\ \vdots \\ \delta \rho_{n}\\ \delta {\dot{\rho }}_{0}\\ \delta {\dot{\rho }}_{1}\\ \vdots \\ \delta {\dot{\rho }}_{n} \end{array}\right]$$

### 5.3. Visual Odometry

The visual odometry estimate vector $x_{c}$ consists of rotations $r_{c}$ and translations $t_{c}$ (where the subscript *c* stands for the camera) that can be represented as:

(19) $$x_{c}={\left[\begin{array}{cc} r_{c} & t_{c} \end{array}\right]}^{T}$$

In fact, the camera and the land vehicle coordinate frames are not the same. Thus, the rotations and the translations of the camera should be transformed into the vehicle coordinate frame following Equations (20) and (21).

(20) $$r_{c_{b}}=R_{c}^{b}r_{c}R_{b}^{c}$$

where $R_{c}^{b}$ is the matrix used for the rotation from the camera to the body frame, $R_{b}^{c}$ the rotation matrix from the body frame to the camera frame and $r_{c_{b}}$ represents the rotated $r_{c}$ into the body frame. Likewise, the translation should also be rotated into the body frame as follows:

(21) $$t_{c_{b}}=R_{c}^{b}\left(t_{c}+r_{c}l_{b}^{c}-l_{b}^{c}\right)$$

where $l_{b}^{c}$ denotes the lever arm from the camera to the body frame.

### 5.4. Tightly-Coupled GNSS/Vision

After applying our multipath mitigation algorithm, there are still a few GNSS satellites (mostly less than four) that are available. Theoretically, at least four satellites are needed to compute the GNSS-only navigation solution [37]. This situation leads us to make a choice of the tightly-coupled GNSS/vision Kalman filter since even with fewer than four satellites, it is possible to use the raw GNSS measurement to improve the efficiency of vision-based navigation.

The system model used is given by:

(22) $$\dot{x}\left(t\right)=F\left(t\right)x\left(t\right)+G\left(t\right)w\left(t\right)$$

where:

x and F are the state vector and the dynamics matrix, respectively;
G represents the shaping matrix;
*w* is a vector of zero-mean, unit variance white noise.

Thus, the dynamic system is expressed in the form:

(23) $$\dot{x}\left(t\right)=F\left(t\right)x\left(t\right)$$

The dynamic matrix is approximately constant between image frames. Its equivalent in discrete-time is called the state transition matrix $\Phi_{k,k+1}$ that converts the state from epoch *k* to k+1, given by:

(24) $$\Phi_{k,k+1}=\exp\left(F\Delta t\right)$$

Given that the Taylor series [62] of the exponential function is computed as $\exp\left(A\right)=I+A+\frac{A^{2}}{2}+\frac{A^{3}}{6}+\ldots$, the transition matrix in Equation (24) becomes:

(25) $$\Phi_{k,k+1}=I+F\Delta t+\frac{F^{2}\Delta t^{2}}{2}+\frac{F^{3}\Delta t^{3}}{6}+\ldots$$

The state vector incorporates the vehicle position, velocity, acceleration, attitude and the receiver clock as shown in Equation (26).

(26) $$x={\left[\begin{array}{ccccccccccccc} \phi & \lambda & h & v & a & p & \dot{p} & r & \dot{r} & A & \dot{A} & cdt & c\dot{dt} \end{array}\right]}^{T}$$

where:

ϕ, λ and *h* represent the position components;
*v* and *a* stand for speed and acceleration, respectively;
*A*, *p* and *r* are the azimuth, pitch and roll respectively;
$\dot{\left(•\right)}$ represents their corresponding rates.

The error states are:

(27) $$\delta x={\left[\begin{array}{ccc} \delta x_{vehicle} & \delta x_{vision} & \delta x_{clock} \end{array}\right]}^{T}$$

which is equivalent to:

(28) $$\delta x={\left[\begin{array}{ccccccccccccc} \delta \phi & \delta \lambda & \delta h & \delta v & \delta a & \delta p & \delta \dot{p} & \delta r & \delta \dot{r} & \delta A & \delta \dot{A} & \delta cdt & \delta c\dot{dt} \end{array}\right]}^{T}$$

and the receiver clock model is given by:

(29) $$\delta x_{clock}\left(t\right)=\left[\begin{array}{cc} 1 & \Delta t\\ 0 & 1 \end{array}\right]\delta x_{clock}\left(t-1\right)+\mu_{clk}$$

where $\delta x_{clock}={\left[\begin{array}{cc} \delta cdt & \delta c\dot{dt} \end{array}\right]}^{T}$

The prediction of the state and the corresponding error covariance matrix are given by Equations (30) and (31), respectively.

(30) $${\hat{x}}_{k+1}^{-}=\Phi_{k,k+1}{\hat{x}}_{k}^{+}$$

(31) $$P_{k+1}^{-}=\Phi_{k,k+1}{\hat{P}}_{k}^{+}\Phi_{k,k+1}^{T}+Q_{k}$$

where the superscript minus and plus respectively denote the state before and after an update; and $Q_{k}$ is the process noise matrix.

The state and the corresponding error covariance matrix after the update stage are given in Equations (32) and (33).

(32) $${\hat{x}}_{k}^{+}={\hat{x}}_{k}^{-}+K_{k}\left(z_{k}-H_{k}{\hat{x}}_{k}^{-}\right)$$

(33) $$P_{k}^{+}=\left(I-K_{k}H_{k}\right)P_{k}^{-}$$

where:

H denotes the design matrix, which is the derivative of the measurements with respect to the states;
$K_{k}=P_{k}^{-}H_{k}^{T}{\left(H_{k}P_{k}^{-}H_{k}^{T}+R_{k}\right)}^{-1}$ represents the Kalman gain.

The term in the Kalman filter state update in the brackets (in Equation (32)) is called the innovation. It is defined as:

(34) $$v_{k}=z_{k}-H_{k}{\hat{x}}_{k}^{-}$$

The innovation is interpreted as the new information brought from the measurements and is used in the context of statistical testing and reliability [63] to prevent measurement errors from corrupting the solution.

#### 5.4.1. GNSS Observables

Because tight coupling is used, it is necessary to estimate the receiver clock offset and clock drift in addition to the position and velocity states. Having this in mind, the GNSS measurements that fit the Kalman filter are the error in the range and the error in the range rate (to each satellite), expanded at the current estimate of the states. This is expressed as:

(35) $$\delta z_{Gnss}=\left[\begin{array}{c} \rho_{i}-\left({\hat{\rho }}_{i}+cdt\right)\\ {\dot{\rho }}_{i}-\left(\hat{\dot{\rho_{i}}}+c\dot{dt}\right)\\ \vdots \end{array}\right]$$

where the parameters are defined as in Section 5.1.

#### 5.4.2. Vision Observables

**Figure 11:**
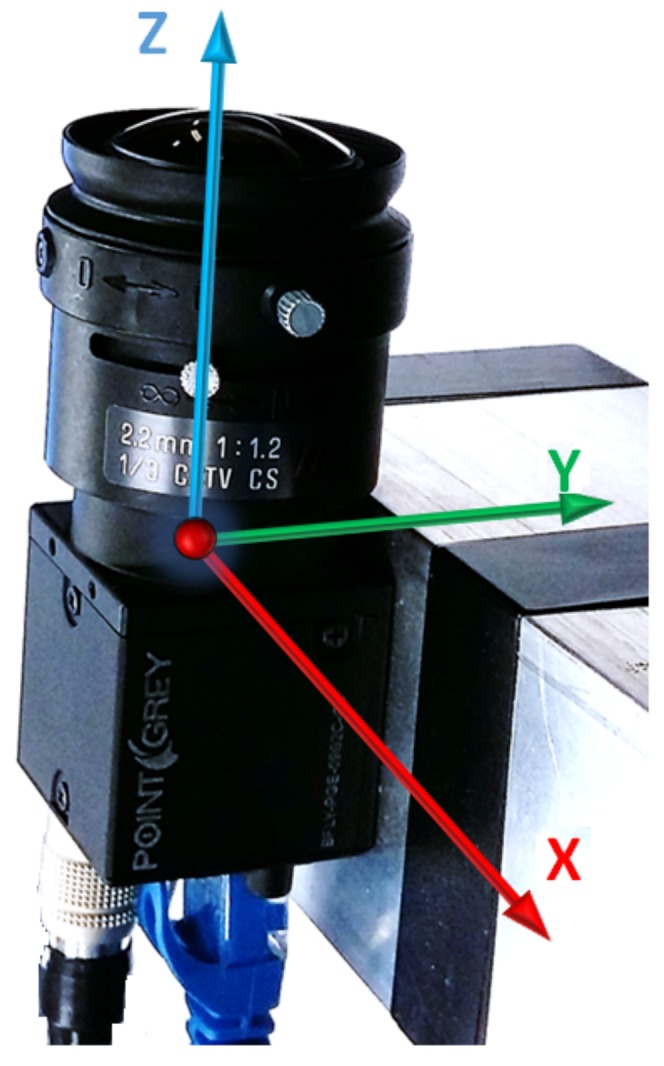
Upward-pointing camera coordinates system.

The vision observables that are added to the tight integration filter are the rotation and translation around and along the axes, as depicted in Figure 11.

However, as presented in [42], it is also possible to consider the distance and azimuth rate as the vision measurement, by considering the elapsed time between corresponding frames. The vision system measurement vector is given as:

(36) $$\delta z_{Vision}=\left[\begin{array}{c} r_{c_{b},k}\\ t_{c_{b},k} \end{array}\right]$$

where *k* corresponds to the image frame number.

### 5.5. State Estimation and Data Integration

The filter is initialized by the first epoch GNSS-only solution. Monocular visual odometry directly provides the attitude and the translation of the platform. The scale factor can be initialized by considering the two first image frames. In this case, the baseline corresponding to the two locations is synthesized and measured. This information can then be used to derive the depth information in a similar way used for stereo vision-based depth estimation [26,29].

The scale ambiguity was resolved in [26] by integrating the GPS carrier phase measurements with the vision system. In our case, we are using pseudorange and pseudorange rate as the measurement. For simplicity and illustration, let us consider a case where the vehicle moves forward without any rotation (translation only). The vehicle position vector and feature points ranges [26] are related as:

(37) $$H_{j}\;\cdot \left[\begin{array}{c} \Delta r\\ s_{j} \end{array}\right]=0_{2\times 1}$$

where:

$0_{2\times 1}$ denotes a 2×1 vector of zeros;
j=1,…,M, with *M* the total number for features;
$H_{j}$ is the matrix defining the homogeneous coordinates of the feature points from consecutive image frames;
Δr is the position change vector between consecutive frames;
$s_{j}$ denotes the unknown range.

On the other hand, given the pseudorange defined as in Equation (12), the changes in pseudorange measurements between two consecutive images are related to Δr:

(38) $$\Delta \rho_{j}=\Delta r+c\Delta t+\Delta \mu$$

Combining Equations (37) and (38) defines a system of linear equations that can be used to unambiguously resolve the position changes, as well as the range estimates [26]. This procedure can be used for the vision system ranges and position changes’ initialization. The assumption here is that the vehicle frame is aligned with the world frame.

Once the system is initialized, the scale factor can be estimated based on the actual and previous estimated platform position. Thus, we have:

(39) $${\hat{s}}_{k}=\sqrt{{\left({\hat{x}}_{k}-{\hat{x}}_{k-1}\right)}^{2}+{\left({\hat{y}}_{k}-{\hat{y}}_{k-1}\right)}^{2}+{\left({\hat{z}}_{k}-{\hat{z}}_{k-1}\right)}^{2}}$$

where:

$\left(\hat{x},\hat{y},\hat{z}\right)$ represent the estimated platform’s position.

**Figure 12:**
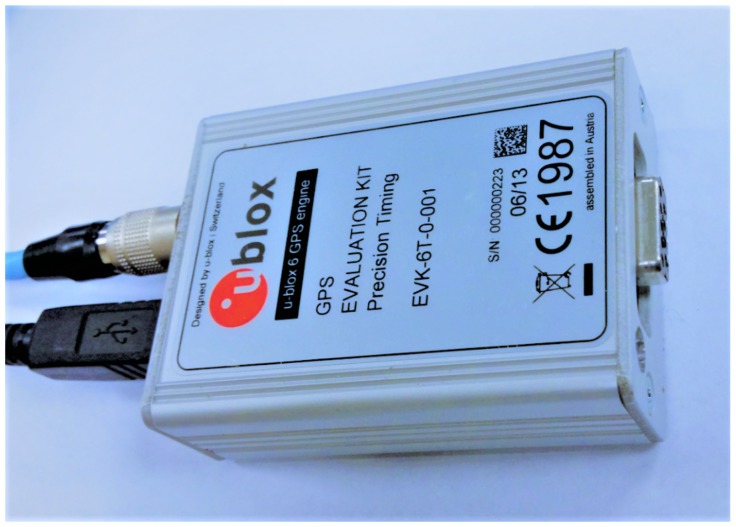
The consumer-grade GNSS receiver used.

The filter is implemented with prediction performed at a rate of 10 Hz. This means that at an interval of 100 ms, the system checks if a new GNSS observation or visual odometry estimated parameter is available. As for the method applied in [42], the vision system’s updates are processed at 10 Hz, and the update from the GNSS receiver (depicted in Figure 12) is performed at 1 Hz.

In the implementation presented herein, the vision measurements are integrated in the form of a filter update. The platform’s attitude is obtained by using the velocity estimates, i.e., by taking the arctangent of the ratio between the velocities in the east and north directions, respectively, for the azimuth for example.

## 6. Experiment and Results

The performance of the proposed approach is demonstrated in this section via a real-world experiment conducted in downtown Calgary.

### 6.1. Hardware

**Figure 13:**
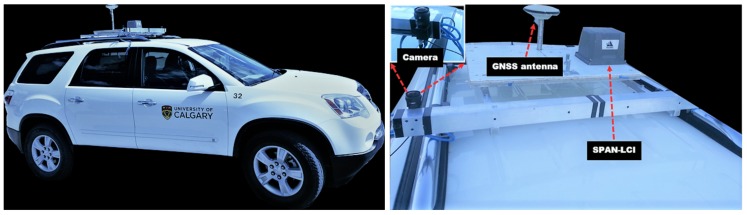
Experiment setup. Left: the used vehicle with the mounted equipment; Right: top view of the vehicle.

The experimental system consisted of a camera synchronized with a u-blox 6T GPS receiver rigidly mounted on the top of the car and driven around downtown Calgary (Alberta, Canada). The camera has a field of view of $90^{\circ }$, and images with a resolution of 1288×728 were taken at a frequency of 10 frames per second. A NovAtel SPAN^®^ LCIsystem that includes a GNSS receiver and an LCI inertial navigation system was used as the reference system. The reference solution is generated post-mission, and its accuracy is at the decimetre-level or better [14]. Figure 13 shows the test vehicle, as well as a zoomed-in view of the equipment mounted on the roof of the vehicle.

The NovAtel GNSS module, as well as the consumer-grade GNSS receiver and the computers that record the images and GNSS data are inside the vehicle and not shown.

**Figure 14:**
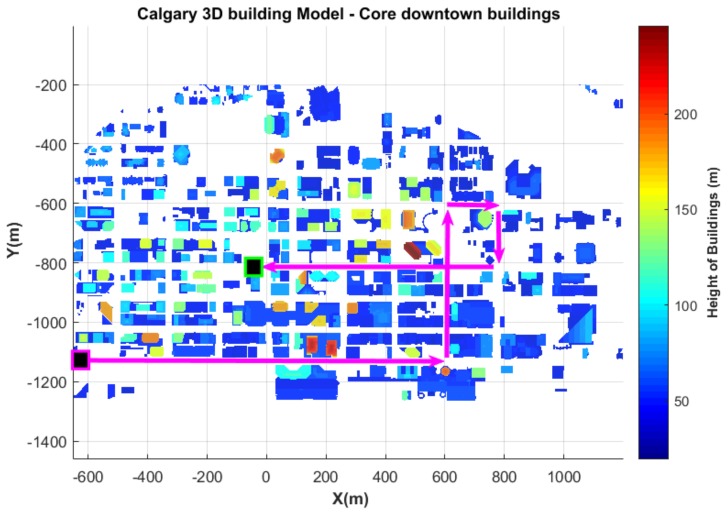
Data collection path travelled. The vehicle starts at the bottom black box (with purple edges), travels following the path indicated with the arrows and stops at the top box (with green edges).

The vehicle was driven around areas with tall buildings as well as areas with short buildings (and with buildings on one side of the road). The travelled path and the buildings’ height in downtown Calgary are shown in Figure 14. The buildings’ height is obtained from the commercially available 3D building model presented in [17,22,64]. Since the camera is pointed upwards, both the NLOS mitigation algorithm and the visual odometry are influenced by the building height. Tall buildings captured by the camera mean that the path between the satellites and the GNSS antenna is highly obstructed. This suggests that the GNSS may perform poorly. Inversely, high buildings in an image provide more feature points to be detected in successive images and, thus, leads to more accurate visual odometry.

The hardware specifications of the equipment used in this paper are provided in Table 1.

where:

VFOV stands for Vertical Field of View;
$r_{i\;(i=1,2,3)}$ and $t_{j\;(j=1,2)}$ are the radial lens and the tangential distortions obtained from the calibration matrix as defined in Section 3.1.

### 6.2. Results and Interpretations

Our results will be evaluated by using the outputs of three integration approaches that will be compared with the reference trajectory:

- The GNSS-only navigation solution: For this, the PLAN-nav (University of Calgary’s module of the GSNRx™ software receiver) was used. As with most consumer-grade receivers, it uses local level position and velocity states for its GPS-only Kalman filter. In the results, this solution is referred to as GNSS-KF;
- The tightly-coupled GNSS/vision solution: The Line Of Sight (LOS) satellites are first selected. Then, this solution tightly couples the vision-based relative motion estimate to the GNSS. In the results, this is referred to as Tightly-Coupled (TC) GNSS/vision;
- The loosely-coupled GNSS/vision solution integrates measurements from the vision system with the GNSS least squares PVTsolution obtained by using range and range rate observations. Both systems independently compute the navigation solutions, and they are integrated in a loosely-coupled way. This means that if one of the system is unable to provide the solution (e.g., GNSS), then no update from that system is provided to the integration filter. This solution will help to clearly see how beneficial the proposed integration method is, especially when there are fewer than four (LOS) satellites. We refer to this as Loosely-Coupled (LC) GNSS/vision in the text. More details on integration strategies can be found in [10,65].

**Figure 15:**
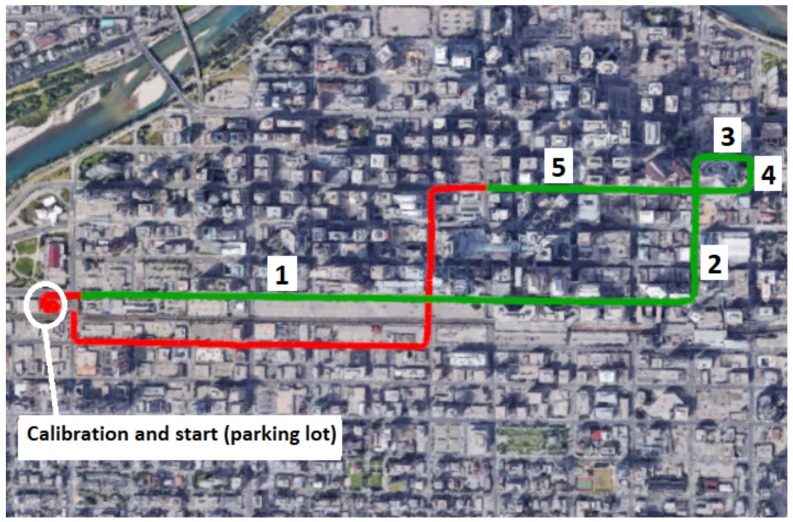
Reference path. Red: the entire travelled path when data were collected; green: the considered path in this paper. 1, 2, 3, 4, 5: portions of the travelled path before turns.

In this work, only GPS L1 C/A pseudoranges and range rates are used. The entire path travelled during the data collection and the path considered for evaluating our proposed method are depicted in Figure 15 (in red and green, respectively, connecting Points 1, 2, 3, 4 and 5, as indicated by the arrows in Figure 14).

The reason why we choose the green path for our experiment has been discussed in Section 6.1 and is shown in Figure 14.

After the equipment calibration (in the circled area depicted in Figure 15), the vehicle stayed stationary for a few minutes before the experiment started. As observed in [42], at the beginning of the experiment, the initial heading of the GNSS/vision integrated solution can be very inaccurate due to the fact that the absolute heading is not available when the car is static. However, once the vehicle starts moving, the heading is quickly recovered.

**Figure 16:**
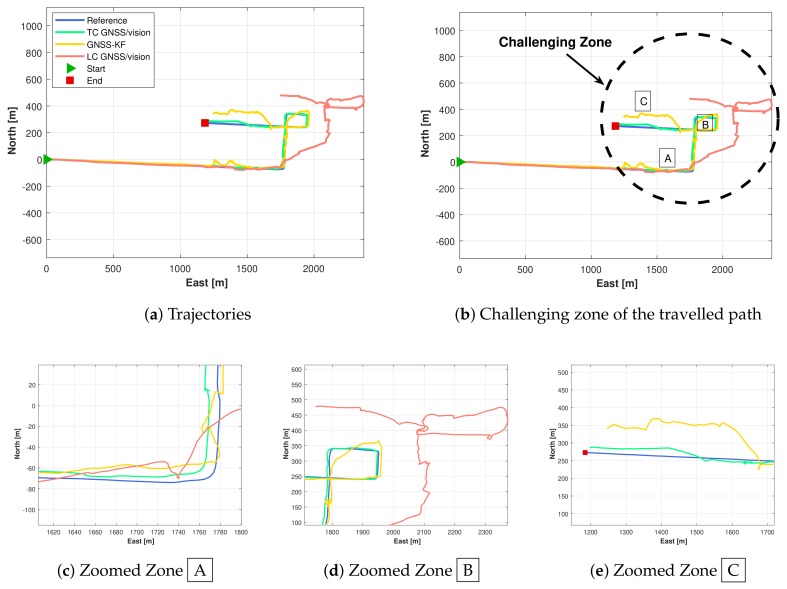
Estimated positions results. (**a**) Estimated position; (**b**) highlighted challenging zones; (**c**) zoomed challenging Zone 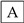; (**d**) zoomed challenging Zone 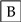; (**e**) zoomed challenging Zone 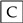. TC, Tightly-Coupled; LC, Loosely-Coupled.

Figure 16 shows the reference (true) trajectory and the position estimation for GNSS-KF, Tightly-Coupled GNSS/vision (TC GNSS/vision), as well as the Loosely-Coupled GNSS/vision (LC GNSS/vision) integrated navigation.

**Figure 17:**
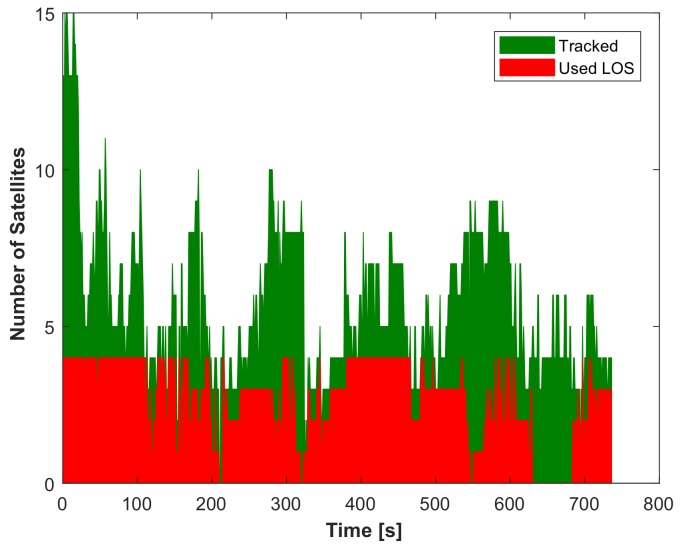
Number of tracked and LOS satellites over time.

**Figure 18:**
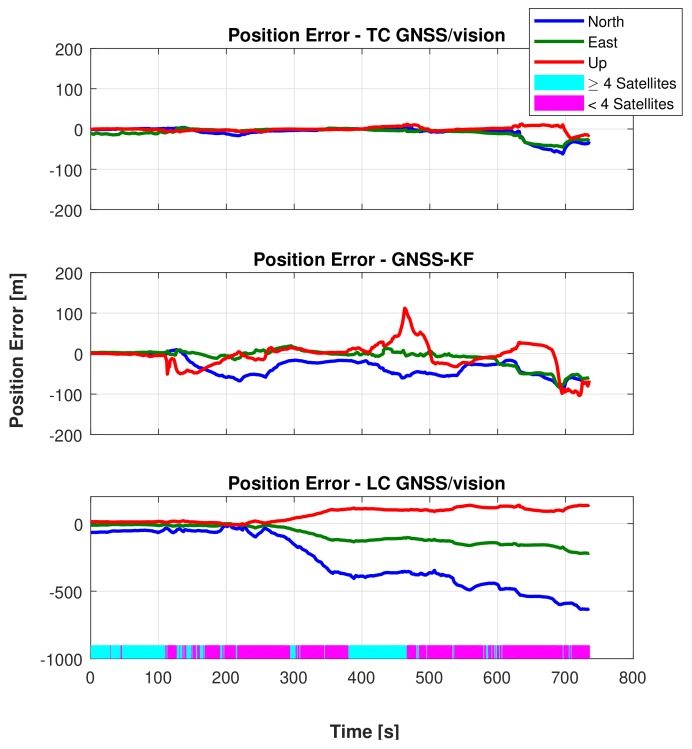
Comparison of position error characteristics between GNSS-KF and the proposed TC GNSS/vision.

From the plotted trajectory, it can be seen that the presented integration approaches closely follow the reference trajectory during the portion of the path where buildings are low (Path 1, shown in Figure 15). During this portion of the trajectory (mostly with one side of the road non-obstructed), after excluding NLOS satellites, four satellites remain (see Figure 17), and the TC GNSS/vision integrated solution is relatively accurate. However, the second portion of the trajectory that contains a mixture of short and tall (≥100 m tall; see Figure 14) buildings (Paths 2, 3, 4 and 5 in Figure 15) is a bit more challenging for two reasons: (i) the first reason is the height of the buildings. Downtown Calgary buildings in general range from 20–50 stories with roads about 15 m wide [64], and even if measurements pass the NLOS selection process, the multipath effect is expected to be large. (ii) The second reason is that the vehicle “stops”. There was severe traffic during this portion of the test. The vehicle was frequently stopping and starting. Since feature points were detected on the sky (and “non-static” clouds), this influenced the accuracy of the visual odometry outputs. When these two phenomena were occurring at the same location, the integrated solution was degraded as shown in Figure 16 and Figure 18. From around Zone 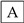, to the end of the travelled path, there are less than four satellites (after NLOS exclusion) 75.8% of the time. For this reason, the LC GNSS/vision integration, which in this situation relies on the vision system only, performs poorly, while the proposed TC GNSS/vision closely follows the reference solution.

**Figure 19:**
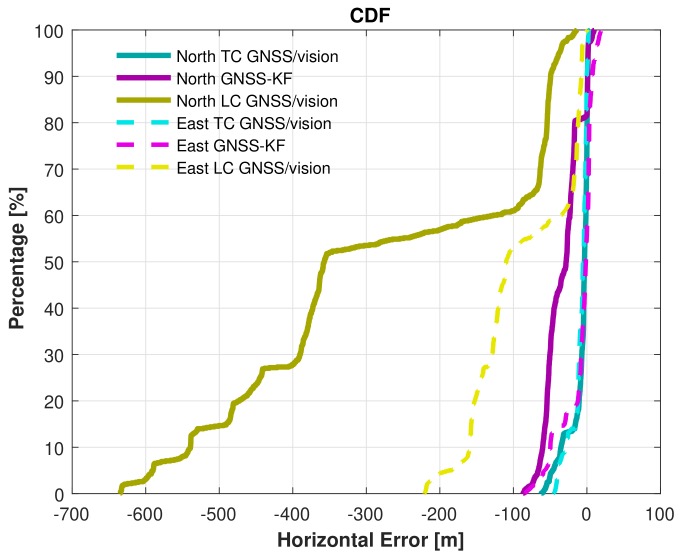
Cumulative distribution functions of the horizontal errors.

Overall, the proposed method provides consistent accuracy over the travelled path with a few exceptions at the turns. At Zones 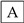 and 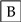, we can observe that the proposed method does not drift too much while the GNSS-KF provides poor solutions. From Figure 18, it is seen that the solution obtained from the LC GNSS/vision outputs larger error compared to the two other approaches. In Zone 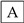 (Figure 16), after NLOS satellites exclusion, there are constantly less than four satellites (Figure 16). As a result, GNSS-aiding is not available, and the obtained navigation solution is poor. The Cumulative Distribution Functions (CDF) of the horizontal errors are provided in Figure 19.

From Figure 19, considering the north direction, the TC GNSS/vision performs better than the GNSS-KF approach 82% of the time and 100% of the time when compared with the LC GNSS/vision. In the east direction, the proposed method performs better than the GNSS-KF 20% of the time and 99% of the time than the LC GNSS/vision. The performance of the proposed method over the LC GNSS/vision indicates the benefit of coupling the LOS satellites with the visual odometry. It can clearly be seen that when the GNSS PVT solution is not available (which happens 65.5% of the time during the data collection), tight coupling of the small number of available GNSS observations is still able to improve the accuracy compared to using no GNSS observations (in the loose coupling case). The proposed method provides a significant improvement over the GNSS-only solution, demonstrating the benefit of fusing visual odometry with the GNSS solution.

**Figure 20:**
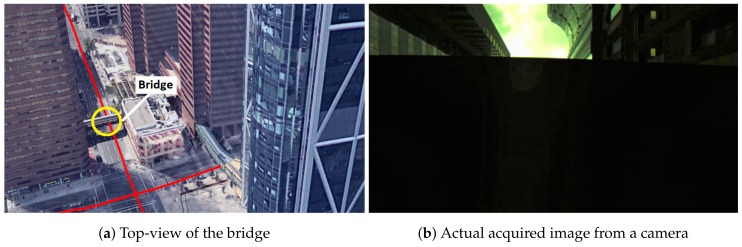
Bridge causing position errors.

Returning back to Figure 16, Zone 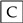 depicts a straight moving zone where both TC GNSS/vision and GNSS-KF provide a poor position estimation. This case, although not alarming for the proposed method, requires further investigation. The two reasons mentioned previously that cause poor integrated solutions occur at this location. Due to a traffic jam, the vehicle stopped under a bridge where LOS satellites were not observed, and the visual odometry was very poor, caused by the fact that very few feature points were detected (the very dark captured images are shown in Figure 20b).

**Figure 21:**
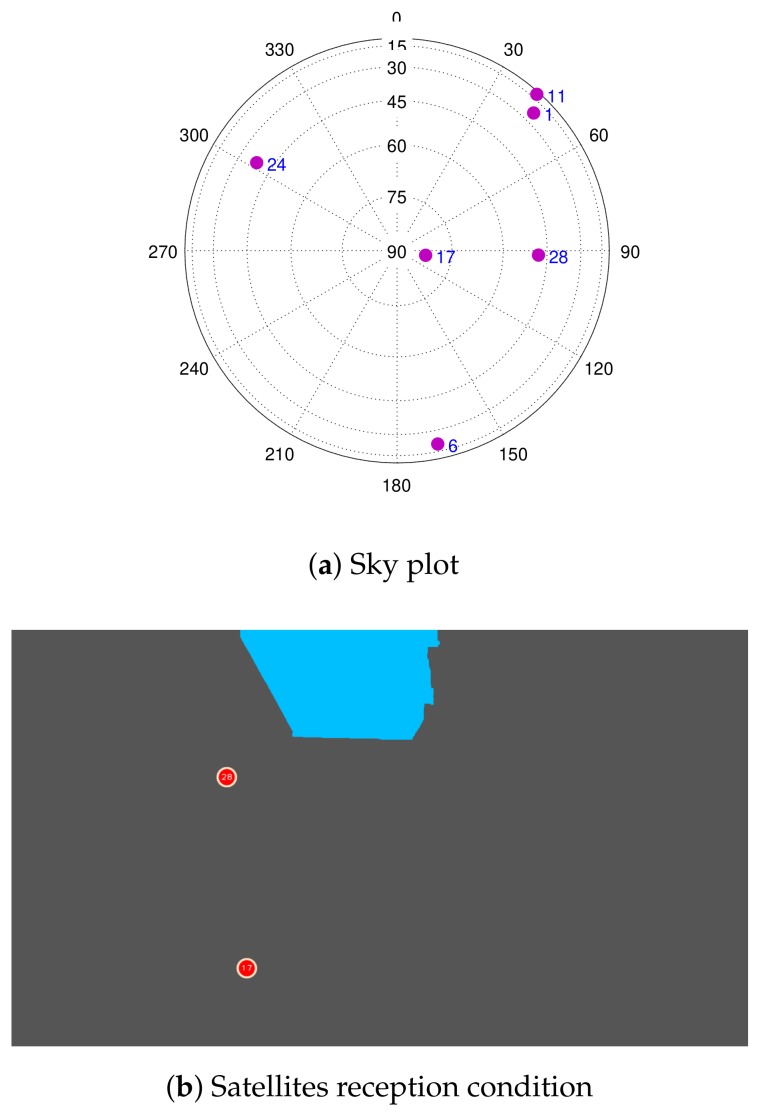
Sky plot of the GPS constellation at Epoch 413179 and its reception conditions.

In this situation a GNSS/vision/INS integrated system, for example (or GNSS/vision/ “appropriate sensor”), would be beneficial. The GPS constellation at this time is shown in Figure 21a. Due to the camera’s FOV, the high elevation satellites that appear to be obstructed by the bridge are in fact tracked, but are excluded from both solutions. This is shown in Figure 21b. The remaining satellites that fall out of the image are considered as obstructed.

**Figure 22:**
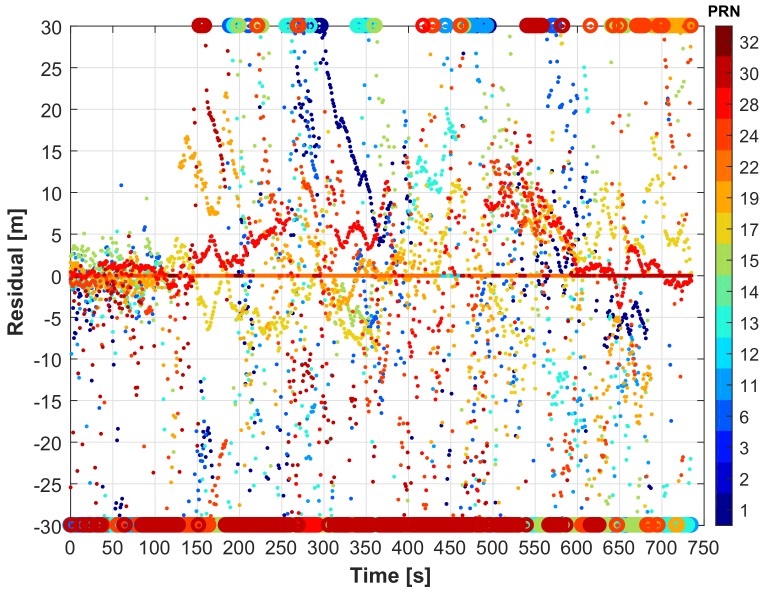
GNSS-only PR residuals.

From the number of the tracked and LOS satellites over time depicted in Figure 17, it can be seen that when the vehicle is stopped under the bridge, although a few satellites were still tracked, no LOS was observed (see Figure 21b and Figure 17, Epochs 628 to 684). To see the performance in terms of the reliability, the residuals of the satellites are shown in Figure 22.

These represent the difference between the actual and the solved measurements and thus indicate the level of agreement of the observations. In open-sky (during the first 100 s), the innovation values are bounded between ±5 m. In the challenging zone, they are larger. The end circle markers in Figure 22 indicate values in excess of ±30 m. By applying statistical testing, satellites with larger innovation values, i.e., the outliers, are detected, and their measurements are prevented from corrupting the solution in the GNSS-KF case. For example, at Epoch 413179 (Figure 21), PRN 19 has a high elevation, but has very large innovation values (Figure 22); thus, its measurements are not used in the GNSS-only case. Even if not the case, our NLOS mitigation method had discarded this specific PRN since the projected satellite falls out of the image “scope” and is considered as obstructed. However, in some cases, satellites with small innovation values can be obstructed and thus degrade the final position solution. PRN17 is a good example of this. PRN17 has a very high elevation and small innovation values, most of the time. Its measurements are thus trusted and used in the GNSS-KF even if obstructed (“slightly” degrading the solution). In this case, the NLOS mitigation algorithm is able to determine this PRN as obstructed, and its measurements are not used, leading to an improved solution. Throughout the test, this occurs with other high satellites that are briefly obstructed.

Surprisingly, as shown in Figure 18, during the first 100 s, the GNSS-KF strategy outperforms the TC GNSS/vision approach in the east direction. The obvious reason behind this is the poor vision performance during this time span (that was close to an open-sky scenario: one side of the road unobstructed, thus, less and bad feature point distribution). However, once the receiver’s antenna is obstructed, the TC GNSS/vision approach becomes more accurate than GNSS-KF.

The statistics of the 2D position errors of the considered path are shown in Table 2.

These statistics are comparable or better than the results obtained in [29] for urban canyon tightly-coupled GNSS/vision (with similar equipment) where a stereo camera system is used instead. This is expected because NLOS satellites are not rejected in this work, which degrades their solution compared to the method presented in this paper.

## 7. Conclusions

This paper presents a tightly-coupled GNSS/vision integration approach. Vehicle motion was estimated by using the visual odometry technique implemented based on feature points detected in successive images acquired by a camera system mounted on the top of the car. An NLOS effect mitigation algorithm tailored for harsh environments and based on LOS satellite selection was also introduced. To improve the LOS satellite selection method, an appropriate image segmentation method using ASF filtering and the flood-fill approach was used to classify image contents into sky and non-sky. Finally, the selected LOS satellites, as well as the vision measurements were integrated in a tightly-coupled manner, taking into account the scale factor since a monocular vision system was used and these types of systems are able to determine the relative translation up to a scale factor only. Results show improvements of 82% and 20% compared to loose coupling of GNSS and visual odometry and to a GNSS-only Kalman filter navigation system, respectively, suggesting that both LOS satellites’ selection and the visual odometry were beneficial. However, some portions of the travelled path were challenging, and the reasons why the algorithm could be influenced were discussed. One of the solutions to stops under bridges where our vision system setup could face challenges (as most of the systems using a camera in a dark environment) could be adding a sensor that is able to provide relative position such as INS or absolute position such as a 3D Building Model (3DBM). In future work, we will use a 3DBM to improve the proposed method. Moreover, instead of employing the commonly-used Kalman filtering technique, the Multiple Model Particle Filter (MMPF), which has proven to better characterize the position errors and estimate the multipath effects [66,67], will be investigated.

## Figures and Tables

**Table 1:** Data collection hardware specifications. VFOV, Vertical Field of View.

**Reference and GNSS**	
SPAN-SE	dual-frequency L1/L2 GPS + GLONASS
Combination	GPS + GLONASS combined with UIMU-LCI
**Camera (Lens Specification, Intrinsic Parameters) and Images**	
Aperture	f/1.2—closed
Focal length	2.2 mm
VFOV (1/3”)	90%
Image resolution	1288×728
Image frame rate	10 fps
Image centre	(643.5, 363.5)
$r_{1}$	−0.26
$r_{2}$	0.08
$r_{3}$	−0.01
$t_{1}$	0
$t_{2}$	0

**Table 2:** Statistics of the considered trajectory.

Estimator	2D rms Error (m)	2D Maximum Error (m)
**GNSS-KF**	39.8	113.2
**LC GNSS/vision**	56.3	402.7
**TC GNSS/vision**	14.5	61.1

## Abbreviations

The following abbreviations are used in this manuscript:

3DBM	3D Building Mode
6-DOF	Six Degrees of Freedom
ASF	Alternate Sequential Filter
CDF	Cumulative Distribution Function
DGPS	Differential Global Positioning System
ECEF	Earth-Centred, Earth-Fixed
EKF	Extended Kalman Filter
FOV	Field of View
GNSS	Global Navigation Satellite Systems
GPS	Global Positioning System
GRD	Geodesic Reconstruction by Dilatation
IMU	Inertial Measurement Unit
KF	Kalman Filter
LOS	Line Of Sight
MMPL	Multiple Model Particle Filter
NLOS	Non-Line of Sight
PnP	Perspective-n-Point
PR	Pseudorange
RGB	Red, Green, Blue (colour space)
SLAM	Simultaneous Localization and Mapping
SVD	Singular Value Decomposition
UAV	Unmanned Aerial Vehicle
UWB	Ultra-Wideband
V2I	Vehicle to Infrastructure
VO	Visual Odometry
